# Laboratory-based surveillance of chronic kidney disease in people with private health coverage in Brazil

**DOI:** 10.1186/s12882-024-03597-9

**Published:** 2024-05-10

**Authors:** Farid Samaan, Rubens Carvalho Silveira, Amilton Mouro, Gianna Mastroianni Kirsztajn, Ricardo Sesso

**Affiliations:** 1 Department of High Complexity Patients, Hapvida NotreDame Intermédica, Rua Hipódromo, 987 - Mooca, São Paulo, SP 03164-140 Brazil; 2 Secretaria de Estado da Saúde de São Paulo, São Paulo, SP Brazil; 3 Instituto Dante Pazzanese de Cardiologia, São Paulo, SP Brazil; 4 Faculdade de Saúde Pública, Universidade de São Paulo, São Paulo, SP Brazil; 5 Disciplina de Nefrologia, Universidade Federal de São Paulo, São Paulo, SP Brazil

**Keywords:** Epidemiology, Prevalence, Information systems in clinical laboratory, Chronic kidney disease, Supplementary health

## Abstract

**Background:**

Although approximately 25% of Brazilians have private health coverage (PHC), studies on the surveillance of chronic kidney disease (CKD) in this population are scarce. The objective of this study was to estimate the prevalence of CKD in individuals under two PHC regimes in Brazil, who total 8,335,724 beneficiaries.

**Methods:**

Outpatient serum creatinine and proteinuria results of individuals from all five regions of Brazil, ≥ 18 years of age, and performed between 10/01/2021 and 10/31/2022, were analyzed through the own laboratory network database. People with serum creatinine measurements were evaluated for the prevalence and staging of CKD, and those with simultaneous measurements of serum creatinine and proteinuria were evaluated for the risk category of the disease. CKD was classified according to current guidelines and was defined as a glomerular filtration rate (GFR) < 60 ml/min/1.73 m² estimated by the 2021 CKD-EPI equation.

**Results:**

The number of adults with serum creatinine results was 1,508,766 (age 44.0 [IQR, 33.9–56.8] years, 62.3% female). The estimated prevalence of CKD was 3.8% (2.6%, 0.8%, 0.2% and 0.2% in CKD stages 3a, 3b, 4 and 5, respectively), and it was higher in males than females (4.0% vs. 3.7%, *p* < 0.001, respectively) and in older age groups (0.2% among 18-29-year-olds, 0.5% among 30-44-year-olds, 2.0% among 45-59-year-olds, 9.4% among 60-74-year-olds, and 32.4% among ≥ 75-year-olds, *p* < 0.001) Adults with simultaneous results of creatinine and proteinuria were 64,178 (age 57.0 [IQR, 44.8–67.3] years, 58.1% female). After adjusting for age and gender, 70.1% were in the low-risk category of CKD, 20.0% were in the moderate-risk category, 5.8% were in the high-risk category, and 4.1% were in the very high-risk category.

**Conclusion:**

The estimated prevalence of CKD was 3.8%, and approximately 10% of the participants were in the categories of high or very high-risk of the disease. While almost 20% of beneficiaries with PHC had serum creatinine data, fewer than 1% underwent tests for proteinuria. This study was one of the largest ever conducted in Brazil and the first one to use the 2021 CKD-EPI equation to estimate the prevalence of CKD.

**Supplementary Information:**

The online version contains supplementary material available at 10.1186/s12882-024-03597-9.

## Background

Chronic kidney disease (CKD) is a major global public health problem, as a common condition that is silent in its early stages, preventable, neglected in many places and costly for all health systems [1]. In developed countries, more than 10% of adults have some degree of CKD, and most of this prevalence is increasing [2]. The complications of CKD can be avoided or mitigated with adequate care, and screening and early treatment are cost-effective in the population at risk [3, 4]. Nations with universal health insurance coverage have 5–7% of their budgets consumed by kidney replacement therapy, which is the final phase of care for CKD [5]. Nevertheless, CKD is not a subject of the prioritized programs to combat chronic noncommunicable diseases (NCDs) in many countries, including Brazil [6]. Between 2008 and 2023, the expenditures of the Brazilian Unified Health System (SUS) on outpatient kidney replacement therapy increased from approximately 1.7 to 4.1 billion Brazilian reais (nearly 340 to 820 million US dollars) [7].

Only in theory does Brazil have clinical guidelines for comprehensive care of patients with CKD and specific ministerial ordinance for the operation of health services relevant to this care [8, 9]. In fact, national studies have shown several inadequacies in the early identification of CKD by primary health care and in the population’s access to consultations with a nephrologist and to CKD diagnosis and follow-up tests [10, 11]. Despite these gaps that are highly present in the Brazilian scenario, this country is one of the few places in the world with universal coverage for dialysis and kidney transplantation [12, 13]. Among the public health actions relevant to the secondary prevention of CKD, epidemiological surveillance stands out, especially given the asymptomatic nature of the early stages of this disease and that many of the traditional nephroprotective medications are low-cost and covered by SUS [14–16].

Brazil is a country with a dual health care system in which the public health care system coexists with private health care. Approximately 25% of the Brazilian population has PHC [17]. However, detailed information on programs to combat NCD or care for complex patients in private health care is scarce [18]. The objective of this study was to estimate the prevalence of CKD in individuals with PHC in Brazil.

## Methods

### Study design and population

**Fig. 1:**
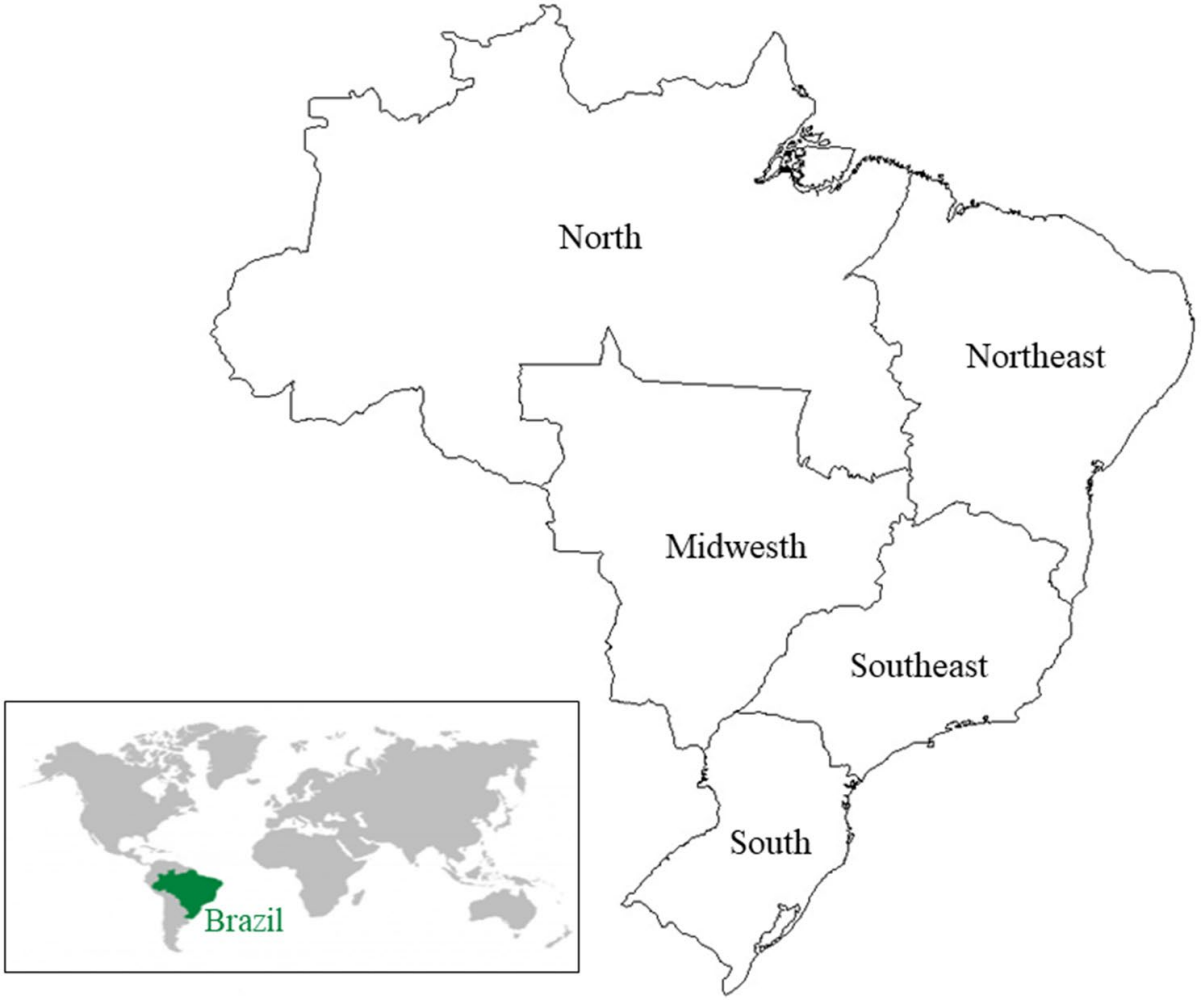
Map of Brazil according to five major regions

This was a retrospective, cross-sectional study based on records of laboratory tests performed between 10/01/2021 and 10/31/2022 in the own laboratory network of two private health companies in Brazil. Together, these companies are national in scope and have a medical assistance portfolio consisting of approximately 8,335,724 beneficiaries [19]. Outpatient serum creatinine and proteinuria tests of individuals from all five regions of Brazil were analyzed. This country has 26 states, in addition to the federal district, and is divided into five large regions (Fig. 1). The exclusion criteria were tests from patients under 18 years of age and those performed in hospital and emergency settings.

The study adhered to the Declaration of Helsinki and Resolution 466/2012 of the Brazilian National Health Council and was approved by the research ethics committee on 11/01/2022 under number 64260622.1.0000.8098. The free and informed consent form was waived because the study was observational, retrospective, noninterventional and large, making it impossible to communicate with all participants. The extraction of information from the database of the laboratories occurred between 12/01/2022 and 04/13/2023.

### Data source, variables, and definitions

Demographic data of the participants (age, gender and collection site) and results of serum creatinine and urinary protein were obtained from laboratory records. In case of patients with two or more measurements of a given test, the lowest value available was used, to avoid overestimation of the prevalence and severity of CKD.

The estimated glomerular filtration rate (GFR) was calculated from serum creatinine, age, and gender using the 2021 CKD-EPI equation [20]. This equation was chosen because it is the most current and it does not require information on ethnicity, as race correction is no longer recommended and because the Brazilian population is highly mixed. CKD was defined as GFR < 60 ml/min/1.73 m² and was classified into stages 3a (45–59 ml/min/1.73 m²), 3b (30–44 ml/min/1.73 m²), 4 (15–29 ml/min/1.73 m²), and 5 (< 15 ml/min/1.73 m²) [21]. All the participating laboratories performed the serum creatinine tests with standardized methods.Proteinuria was assessed categorically. It was present in the case of any of the following: isolated urine albumin/creatinine ratio (ACR) > 30 mg/g, 24-hour albuminuria > 30 mg/24 h, or 24-hour proteinuria > 150 mg/24 h. The categories were mild (ACR < 30 mg/g, albuminuria < 30 mg/24 h, or proteinuria < 150 mg/24 h), moderate (ACR 30–300 mg/g, albuminuria 30–300 mg/24 h, or proteinuria 150–500 mg/24 h), and severe (ACR > 300 mg/g, albuminuria > 300 mg/24 h, or proteinuria > 500 mg/24 h) [21].

The representativeness of the sample in the regions of Brazil was calculated as the proportion between the number of adults who underwent the evaluated tests and the number of beneficiaries within each region. According to the historical series, the annual amount of serum creatinine tests performed is approximately 2,500,000, but the number of proteinuria measurements is less than 100,000.

Therefore, patients with serum creatinine measurements were evaluated for the prevalence of CKD and its stages, and those with simultaneous serum creatinine and proteinuria measurements were evaluated regarding the risk of CKD. According to the CKD heat map of KDIGO 2012 Clinical Practice Guideline (Kidney Disease Improving Global Outcomes), patients were considered to be at low risk when they had GFR ≥ 60 ml/min/1.73 m² and mild proteinuria. They were at moderate risk when they had GFR ≥ 60 ml/min/1.73 m² and moderate proteinuria or GFR 45–59 ml/min/1.73 m² and mild proteinuria. Patients were in the high-risk category for CKD when they had any of the following combinations: GFR ≥ 60 ml/min/1.73 m² and severe proteinuria, GFR 45–59 ml/min/1.73 m² and moderate proteinuria, or GFR 30–44 ml/min/1.73 m² and mild proteinuria. They were at very high-risk when they had GFR 45–59 ml/min/1.73 m² and severe proteinuria, GFR 30–44 ml/min/1.73 m² and moderate or severe proteinuria, or GFR < 30 ml/min/1.73 m² regardless of the level of proteinuria [21].

### Data analysis

Initially, the distribution of participants by demographic variables (gender, age group, and region of Brazil) and laboratory results (GFR and proteinuria) was evaluated, and their respective 95% confidence intervals were calculated. The distribution of patients in the different GFR ranges was assessed according to gender, age, and region of Brazil. The intergroup distribution was assessed using the χ² test. The crude prevalence of CKD by region of Brazil was estimated as the proportion of the number of people with abnormal tests out of the total number of people who underwent that test. A risk map (or heat map) of CKD was drawn by crossing the GFR information and the proteinuria categories. Finally, the prevalence rates were adjusted for age and gender using the direct method, with the total study population as a reference. Statistical analyses were conducted using R version 4.2.1 software.

## Results

**Fig. 2:**
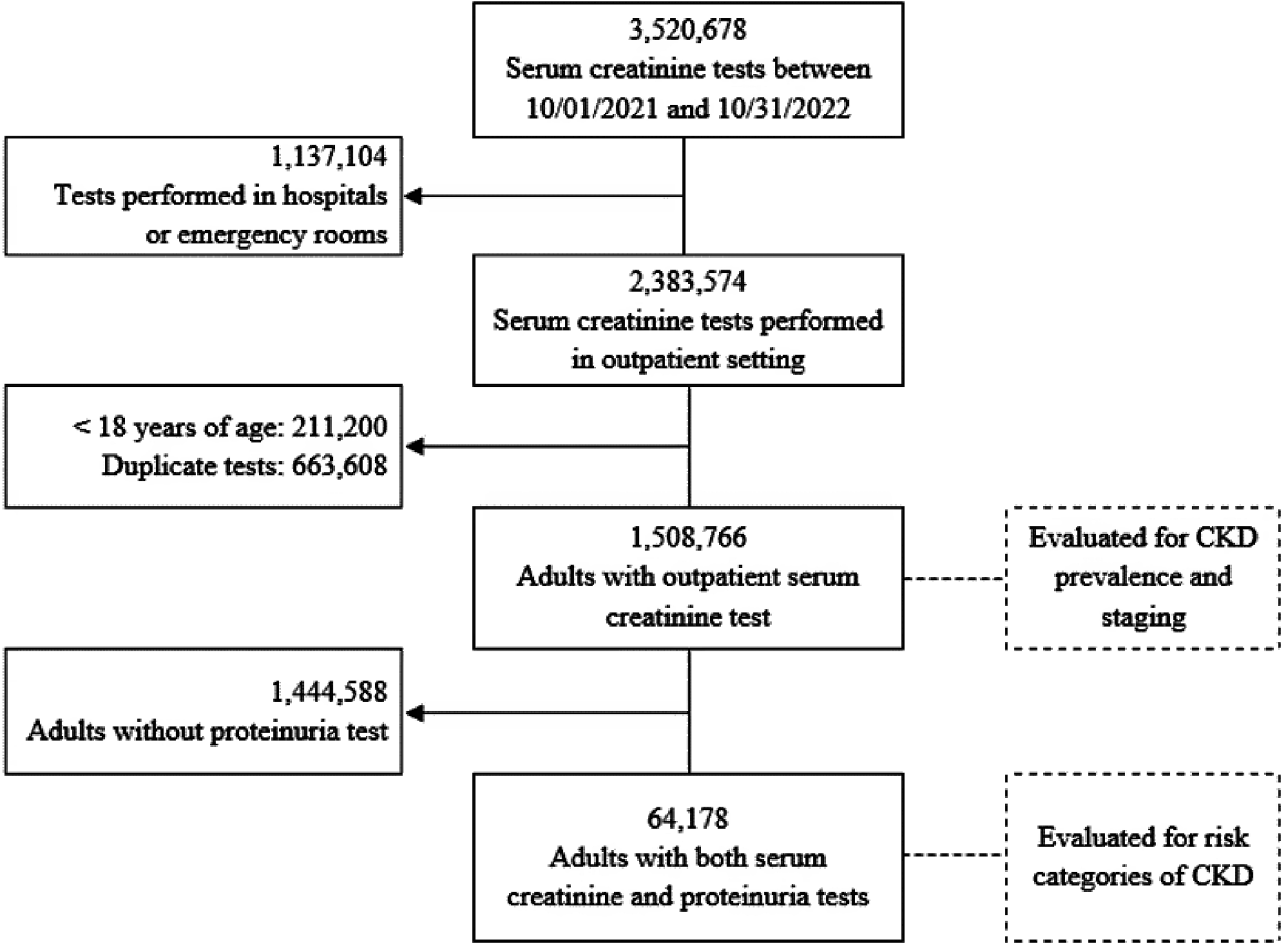
Flowchart of inclusion of study participants. CKD, chronic kidney disease.

Between 1 October 2021 and 31 October 2022, 3,520,678 serum creatinine measurements were recorded. After excluding tests performed in hospitals or emergency rooms (1,137,104), tests performed in people under 18 years of age (211,200), and duplicate tests (663,608), 1,508,766 adults were included for the estimation of CKD prevalence and staging. Among them, 64,178 (4.3%) had results of both serum creatinine and proteinuria, so this was the number evaluated for the risk categories of CKD (Fig. 2).

The adults evaluated by serum creatinine had a median age of 44.0 (33.9–56.8) years, were predominantly female (62.3%), and were from the Southeast region (45.9%). The prevalence of CKD, estimated as the proportion of people with GFR < 60 ml/min/1.73 m², was 3.79% (2.58% with CKD in stage 3a, 0.81% in stage 3b, 0.24% in stage 4, and 0.16% in stage 5). Participants who had both serum creatinine and proteinuria results were 57.0 (44.8–67.3) years old, and 58.1% were female. Their distribution of proteinuria categories was 76.4% mild or absent, 18.1% moderate, and 5.6% severe (Table 1).

**Table 1:** Characteristics of the patients included in the study

Variable	Number	Proportion (%) [95% CI]
Gender		
Male	569,206	37.73 [37.65; 37.80]
Female	939,560	62.27 [62.20; 62.35]
Age group (years)		
18–29	255,997	16.97 [16.91; 17.03]
30–44	524,640	34.77 [34.70; 34.85]
45–59	423,729	28.08 [28.01; 28.16]
60–74	230,436	15.27 [15.22; 15.33]
75 or older	73,964	4.90 [4.87; 4.94]
Region of Brazil		
North	105,365	6.98 [6.94; 7.02]
Northeast	538,083	35.66 [35.59; 35.74]
Midwest	75,053	4.97 [4.94; 5.01]
Southeast	692,055	45.87 [45.79; 45.95]
South	98,210	6.51 [6.47; 6.55]
Range of GFR (ml/min/1.73 m²)		
≥ 60	1,451,552	96.21 [96.18; 96.24]
45–59	38,888	2.58 [2.55; 2.60]
30–44	12,276	0.81 [0.80; 0.83]
15–29	3,652	0.24 [0.23; 0.25]
< 15	2,398	0.16 [0.15; 0.17]
Proteinuria		
Mild	49,010	76.37 [76.03; 76.69]
Moderate	11,586	18.05 [17.76; 18.35]
Severe	3,582	5.58 [5.41; 5.76]

CI, confidence interval. GFR, estimated glomerular filtration rate

Patients with serum creatinine values made up 18.1% of the total beneficiaries with PHC (20.2% in the North, 24.2% in the Northeast, 13.9% in the Midwest, 15.2% in the Southeast, and 19.6% in the South). Patients with both serum creatinine and proteinuria data were 0.8% of the beneficiaries (0.7% in the Northeast, 0.4% in the North, 1.7% in the South, 0.8% in the Southeast, and 0.6% in the Midwest) (Table 2).

**Table 2:** Representativeness of the sample, according to the region of Brazil

Region of Brazil	Number of beneficiaries¹	People with serum creatinine measurement			People with measurement of proteinuria	
		Number	Proportion (%)		Number	Proportion (%)
North	521,609	105,365	20.2		2,276	0.4
Northeast	2,223,483	538,083	24.2		14,685	0.7
Midwest	539,950	75,053	13.9		3,104	0.6
Southeast	4,552,993	692,055	15.2		35,774	0.8
South	501,071	98,210	19.6		8,339	1.7
Total	8,335,724	1,508,766	18.1		64,178	0.8

¹December 2021

**Fig. 3:**
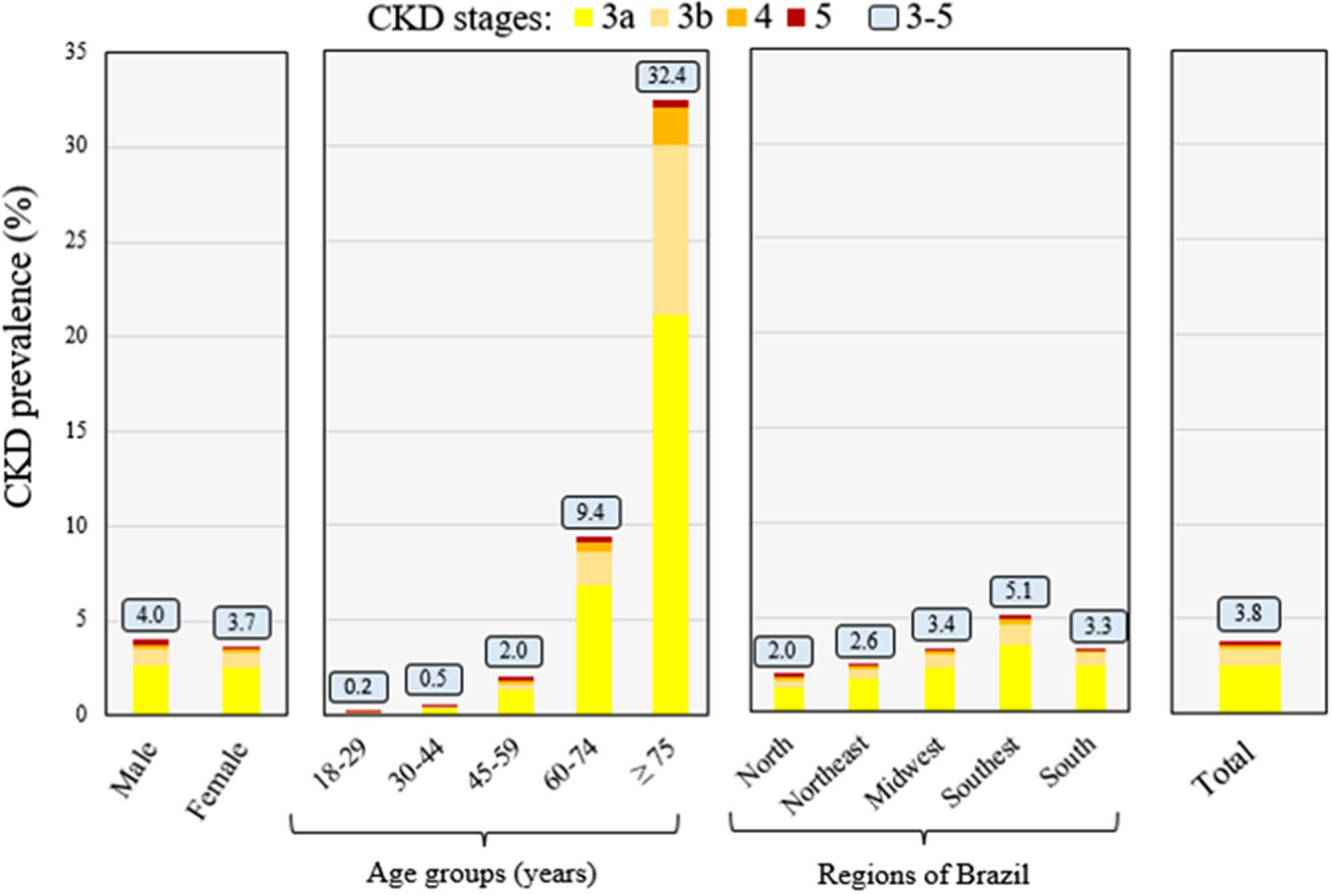
Estimated prevalence of chronic kidney disease according to gender, age, and region of Brazil (*n* = 1,508,766). CKD, chronic kidney disease. Stage 3a, estimated glomerular filtration rate (GFR) 45–59 ml/min/1.73 m². Stage 3b, GFR 30–44 ml/min/1.73 m². Stage 4, GFR 15–29 ml/min/1.73 m². Stage 5, GFR < 15 ml/min/1.73 m²

The proportion of males with GFR < 60 ml/min/1.73 m² was higher than that of females (4.03% versus 3.65%, *p* < 0.001), and the low-GFR proportion increased with age (0.17% among 18-29-year-olds, 0.53% among 30-44-year-olds, 1.98% among 45-59-year-olds, 9.4% among 60-74-year-olds, and 32.4% among ≥ 75-year-olds, *p* < 0.001). The distribution of the total sample in the various GFR ranges (in ml/min/1.73 m²) was 67.47% at ≥ 90, 28.73% at 60–89, 2.58% at 45–59, 0.81% at 30–44, 0.24% at 15–29, and 0.16% at < 15 (Table 3; Fig. 3).

**Table 3:** Distribution of patients in estimated glomerular filtration rate ranges according to gender, age, and region of Brazil (*n* = 1,508,766)

Variables		Estimated glomerular filtration rate, ml/min/1.73 m² (%)						Total	*p* value
		≥ 90	60–89	45–59	30–44	15–29	< 15		
Gender	Male	63.52	32.45	2.66	0.84	0.29	0.24	100.00	< 0.001
	Female	69.87	26.48	2.53	0.80	0.21	0.11	100.00	
Age group (years)	18–29	92.66	7.17	0.07	0.03	0.03	0.04	100.00	< 0.001
	30–44	80.40	19.06	0.31	0.08	0.05	0.09	100.00	
	45–59	61.69	36.34	1.35	0.29	0.16	0.18	100.00	
	60–74	37.67	52.94	6.86	1.71	0.50	0.33	100.00	
	75 and over	14.61	52.99	21.06	8.96	1.97	0.41	100.00	
Region	North	78.82	19.15	1.24	0.41	0.18	0.20	100.00	< 0.001
	Northeast	75.34	22.08	1.71	0.56	0.18	0.14	100.00	
	Midwest	66.66	29.98	2.35	0.66	0.23	0.12	100.00	
	Southeast	60.28	34.61	3.50	1.11	0.31	0.18	100.00	
	South	63.52	33.15	2.43	0.66	0.17	0.07	100.00	
Total		67.47	28.73	2.58	0.81	0.24	0.16	100.00	

The estimated crude prevalence of CKD was highest in the Southeast region (5.10%), followed by the Midwest (3.36%), South (3.33%), Northeast (2.59%), and North regions (2.03%). The age- and gender-adjusted prevalence of CKD was higher in the Midwest region (4.43%), followed by the Southeast (4.21%), South (3.77%), North (3.35%), and Northeast regions (3.02%) (Table 4).

**Table 4:** Median crude and adjusted CKD prevalence estimates by region of Brazil (*n* = 1,508,766)

Region	Age¹ (years)	Male gender (%)	Crude prevalence of CKD (%)	Adjusted prevalence of CKD² (%)
North	40.0 (31.0–50.0)	35.25	2.03	3.35
Northeast	42.0 (32.0–54.0)	35.65	2.59	3.02
Midwest	41.0 (31.0–53.0)	35.32	3.36	4.43
Southeast	47.1 (36.0-60.3)	40.10	5.10	4.21
South	44.0 (33.6–56.0)	36.94	3.33	3.77
Total	44.0 (3.9–56.8)	37.73	3.79	-

¹ Median (interquartile)

² Adjusted for age and gender, using the total number of participants as the standard population

The proportion of males with proteinuria was higher than that of females (26.08% versus 21.87%, *p* < 0.001). There was no progressive increase in the prevalence of proteinuria with age. The distribution of the total sample in levels of proteinuria was 76.37% at mild, 18.05% at moderate, and 5.58% at severe level (Table 5).

**Table 5:** Distribution of patients in levels of proteinuria according to gender, age and region of Brazil (*n* = 64,178)

Variables		Level of proteinuria (%)			Total	*p* value
		Mild	Moderate	Severe		
Gender	Male	73.92	18.87	7.22	100.00	< 0.001
	Female	78.13	17.46	4.40	100.00	
Age group (years)	18–29	71.06	22.80	6.15	100.00	< 0.001
	30–44	75.32	19.58	5.11	100.00	
	45–59	79.84	15.26	4.90	100.00	
	60–74	75.94	17.86	6.20	100.00	
	75 and over	71.64	21.98	6.38	100.00	
Region	North	77.46	17.71	4.83	100.00	< 0.001
	Northeast	89.66	7.94	2.40	100.00	
	Midwest	90.91	7.44	1.64	100.00	
	Southeast	68.96	23.44	7.60	100.00	
	South	79.01	16.79	4.20	100.00	
Total		76.37	18.05	5.58	100.00	

**Fig. 4:**
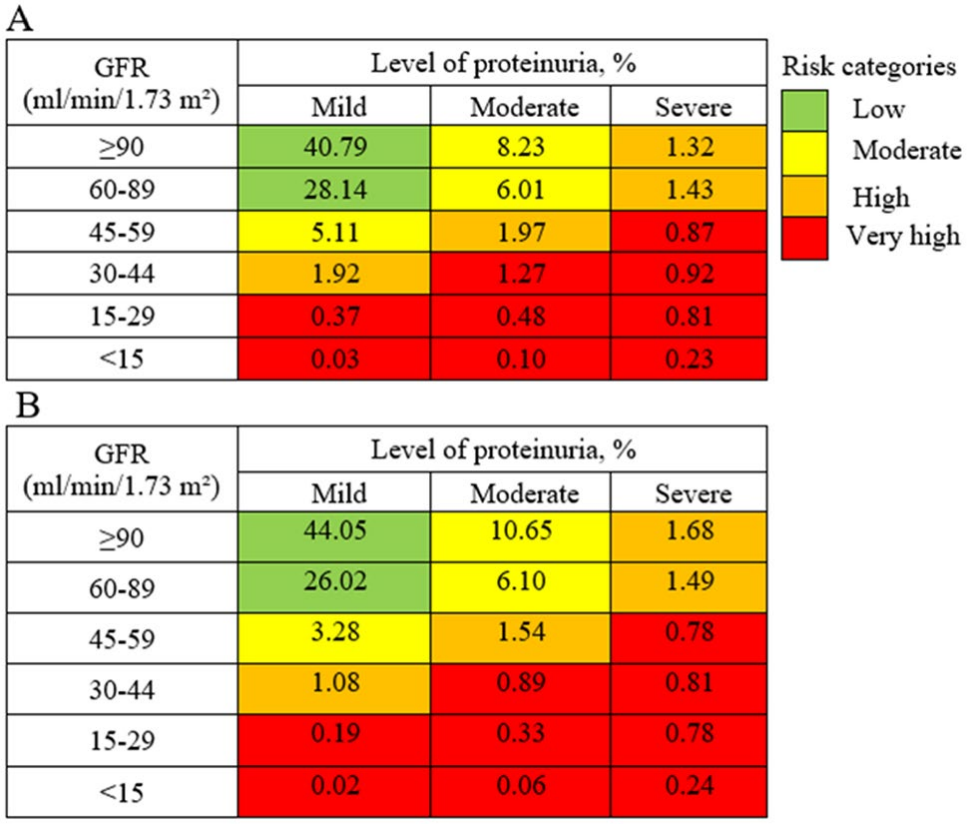
Crude (**A**) and adjusted (**B**) distribution of patients according to the CKD risk map (*n* = 64,178). CKD, chronic kidney disease. A, Gross distribution. B, Distribution adjusted for age and gender. GFR, estimated glomerular filtration rate. Mild proteinuria, albumin/creatinine ratio (ACR) < 30 mg/g or 24-hour albuminuria (24-hour ALB) < 30 mg or 24-hour proteinuria (24-hour PTU) < 150 mg. Moderate, ACR 30–300 mg/g or 24-hour ALB 30–300 mg or 24-hour PTU 150–500 mg. Severe, ACR > 300 mg/g or 24-hour ALB > 300 mg or 24-hour PTU > 500 mg

According to the CKD risk map, the crude prevalence of patients with low, moderate, high and very high-risk categories for the disease was 68.9%, 19.4%, 6.6% and 5.1%, respectively. After adjusting for age and gender, these prevalences were: 70.1%, 20.0%, 5.8% and 4.1%, respectively (Fig. 4).

## Discussion

The present study showed that the prevalence of CKD in beneficiaries with PHC in Brazil was 3.8%, according to the GFR estimated by the 2021 CKD-EPI equation. After adjusting for age and gender, this prevalence was higher in the Midwest and Southeast and lower in the North and Northeast of Brazil. While 18.1% of the beneficiaries were tested for serum creatinine, only 0.8% underwent measurement of proteinuria. Among them, approximately 10% were at high or very high-risk categories of CKD.

In recent decades, several studies have estimated the prevalence of CKD (Table 6 S) [22–44]. The great variability of results (0.8–32.5%) is due, among other factors, to the population profile (age, comorbidities), the data sources (telephone survey, disease codes, screening programs, population-based studies, laboratory databases), and the definitions of CKD used (reduced GFR with or without altered proteinuria).

The estimated prevalence of CKD of 3.8% found in the present study was higher than that found in studies based on telephone surveys (1.4%) [22, 41] and by registering disease codes (0.8%) [29]. This might have been due to the silent nature of the early stages of CKD, which leads to low awareness among the population about the disease and identification failures of it by health professionals [35]. This fact reinforces the need for screening for CKD in people at risk by measuring serum creatinine and proteinuria [8, 9, 21].

On the other hand, our prevalence of 3.8% was lower than the estimates of CKD prevalence from screening programs (8.9–32.5%), which generally include individuals with risk factors for the disease, such as hypertension, diabetes mellitus, and cardiovascular diseases [25, 26, 28, 33, 35, 36, 38, 39]. Another explanation for the lower prevalence of CKD found in this study might be the fact that we enrolled individuals with PHC, who might have a lower age and burden of comorbidities and better socioeconomic conditions, since they are mostly economically active people with a corporate-type PHC. Unfortunately, detailed information on the comorbidity profile of our population was not available.

When compared to studies based on disease code registration and screening programs, it is possible that population-based studies [34, 37, 40, 42, 43] and those based on laboratory databases [23, 24, 27, 29–32, 44] can include individuals with lower disease burdens. Even so, the prevalence of CKD found in this study (3.8%) was lower than that in previous studies of the same nature (5.6–12.4%). Possible explanations would be the lower mean age of the participants in our study and the use of the 2021 CKD-EPI equation rather than the 2009 equation. In fact, older age is an important risk factor for reduced GFR, and the 2021 CKD-EPI equation may overestimate the GFR by 3–5 ml/min/1.73 m² when compared to the 2009 CKD-EPI equation [21, 45, 46]. In our population, when the 2009 CKD-EPI equation was applied, the prevalence of CKD was estimated at 5.1% (Table 7 S). In addition, the definition of CKD used in this study considered only reduced GFR, as a very small proportion of people in Brazil are routinely tested for proteinuria. This fact constitutes a serious flaw in the implementation of the line of care for CKD in our country, which has already been pointed out by previous national studies [10, 11]. Thus, participants with preserved GFR and present proteinuria were not counted as having CKD.

In our study, the estimated prevalence of CKD was slightly higher in males than in females. On this matter, the previous reports show discordant results. Women could have more CKD than men due to their smaller nephron mass and greater chance of early diagnosis, since in many cultures women are more conscientious about their health than men [23, 25, 28, 31–33, 37, 43–45, 47–49]. On the other hand, men might have a higher prevalence of CKD due to their higher frequency of hypertension and proteinuria [24, 26, 39].

The regional differences found after adjustment for age and gender could not be analysed in detail due to the unavailability of information on the patients’ morbidities. It might be that individuals from the North and Northeast regions of Brazil have a lower prevalence of CKD due to the lower prevalence of hypertension, diabetes, and obesity, as shown in a national telephone survey [50].

The result of 24% of patients with proteinuria and 10% of patients at high or very high-risk categories of CKD found in this cohort is higher than the estimative of previous studies [10, 35]. Possibly patients of our study with proteinuria test requests were sicker than the population with serum creatinine dosages. In fact, the crude prevalence of high or very high-risk categories of CKD was higher than the age- and gender adjusted prevalence.

Some limitations of this study should be acknowledged, such as the unavailability of information on the participants’ comorbidities, the impossibility of using two serum creatinine results in the same individuals to confirm the diagnosis of CKD, and the noninclusion of proteinuria measurement in the assessment of the prevalence of CKD. Nevertheless, the importance of this study lies in its size (one of the largest ever conducted in Brazil), in the national representativeness of individuals with PHC (approximately one-fifth of the beneficiaries were tested for serum creatinine), and because it is the first Brazilian study to use the 2021 CKD-EPI equation to estimate the prevalence of CKD.

## Conclusions

In this Brazilian national study of individuals with PHC, we found a prevalence of CKD of 3.8%, a deficiency of proteinuria screening, and approximately 10% of participants with high or very high-risk for such disease. These results provide relevant information on various strata of the Brazilian population with PHC and serve as a basis for planning interventions aimed at early diagnosis and care of CKD.

### Electronic supplementary material

Below is the link to the electronic supplementary material.

Supplementary Material 1

Supplementary Material 2

## Data Availability

The datasets used and/or analyzed during the current study are available from the corresponding author on reasonable request.

## Abbreviations

24-hour ALB: 24-hour albuminuria
24-hour PTU: 24-hour proteinuria
ACR: Urine albumin/creatinine ratio
CI: Confidence interval
CKD: Chronic kidney disease
GFR: Glomerular filtration rate
NCDs: Noncommunicable diseases
PHC: Private health coverage
SUS: Brazilian Unified Health System

## References

[1] Borg R, Carlson N, Søndergaard J, Persson F. The growing challenge of chronic kidney disease: an overview of current knowledge. Int J Nephrol. 2023;2023:9609266. 10.1155/2023/9609266. 36908289 10.1155/2023/9609266 PMC9995188

[2] Kovesdy CP. Epidemiology of chronic kidney disease: an update 2022. Kidney Int Suppl (2011). 2022;12(1):7–11. 10.1016/j.kisu.2021.11.003. 35529086 10.1016/j.kisu.2021.11.003 PMC9073222

[3] Kakitapalli Y, Ampolu J, Madasu SD, Sai Kumar MLS. Detailed review of chronic kidney disease. Kidney Dis (Basel). 2020;6(2):85–91. 10.1159/000504622. 32309290 10.1159/000504622 PMC7154282

[4] Liu HH, Zhao S. Savings Opportunity from Improved CKD Care Management. J Am Soc Nephrol. 2018;29(11):2612–5. 10.1681/ASN.2017121276. 30279275 10.1681/ASN.2017121276 PMC6218872

[5] Jha V, Al-Ghamdi SMG, Li G, Wu MS, Stafylas P, Retat L, Card-Gowers J, Barone S, Cabrera C, Garcia Sanchez JJ. Global Economic Burden Associated with chronic kidney disease: a pragmatic review of medical costs for the Inside CKD Research Programme. Adv Ther. 2023. 10.1007/s12325-023-02608-9. 37493856 10.1007/s12325-023-02608-9 PMC10499937

[6] Saúde Ministérioda. Brasil. Secretaria de Vigilância em Saúde, Departamento de Análise em Saúde e Vigilância de Doenças Não Transmissíveis. Plano de Ações Estratégicas para o Enfrentamento das Doenças Crônicas e Agravos não Transmissíveis no Brasil 2021–2030. Available on: http://bvsms.saude.gov.br/bvs/publicacoes/plano_enfrentamento_doencas_cronicas_agravos_2021_2030.pdf.

[7] Ministério da Saúde. Brasil. DATASUS. Sistema de Informações Ambulatoriais do Sistema Único de Saúde (SIA/SUS). Available on: https://datasus.saude.gov.br/acesso-a-informacao/producao-ambulatorial-sia-sus/.

[8] Saúde Ministérioda. Brasil. Secretaria de Atenção à Saúde. Departamento de Atenção Especializada e Temática. Diretrizes clínicas para o cuidado ao paciente com doença renal crônica – DRC no Sistema Único de Saúde. Available on: https://portalarquivos2.saude.gov.br/images/pdf/2014/marco/24/diretriz-cl--nica-drc-versao-final.pdf.

[9] Ministério da Saúde, Brasil. Portaria nº 1675 de 2018. Critérios para a organização, funcionamento e financiamento do cuidado da pessoa com Doença Renal Crônica - DRC no âmbito do Sistema Único de Saúde. Available on: https://bvsms.saude.gov.br/bvs/saudelegis/gm/2018/prt1675_08_06_2018.html.

[10] Samaan F, Fernandes DE, Kirsztajn GM, Sesso RCC, Malik AM. Quality indicators for primary health care in chronic kidney disease in the public service of a city in the state of São Paulo, Brazil. Cad Saude Publica. 2022;38(2):e00090821. 10.1590/0102-311X00090821. 35319618 10.1590/0102-311X00090821

[11] Samaan F, Gutierrez M, Kirsztajn GM, Sesso RC. Supply/demand ratio for medical consultations, diagnostic tests and chronic kidney disease monitoring in the Brazilian National Health System: a descriptive study, state of São Paulo, Brazil, 2019. Epidemiol Serv Saude. 2022;31(2):e20211050. 10.1590/S2237-96222022000200014. 35830061 10.1590/S2237-96222022000200014 PMC9887954

[12] Assis Buosi AP, Paturkar D, Dias ER, Estorninho MJ, Kolawole O, Ghooi R, Lutchman S. The rights of patients with chronic kidney disease in the World: Legal perspectives and challenges in Brazil, India, Portugal, South Africa, and Nigeria. Contrib Nephrol. 2021;199:322–38. 10.1159/000517722. 34344007 10.1159/000517722

[13] Silva GBD Junior, Oliveira JGR, Oliveira MRB, Vieira LJES, Dias ER. Global costs attributed to chronic kidney disease: a systematic review. Rev Assoc Med Bras (1992). 2018;64(12):1108–16. 10.1590/1806-9282.64.12.1108. 30569987 10.1590/1806-9282.64.12.1108

[14] Burgos-Calderón R, Depine SÁ, Aroca-Martínez G. Population Kidney Health. A new paradigm for chronic kidney Disease Management. Int J Environ Res Public Health. 2021;18(13):6786. 10.3390/ijerph18136786. 34202623 10.3390/ijerph18136786 PMC8297314

[15] Myers OB, Pankratz VS, Norris KC, Vassalotti JA, Unruh ML, Argyropoulos C. Surveillance of CKD epidemiology in the US - a joint analysis of NHANES and KEEP. Sci Rep. 2018;8(1):15900. 10.1038/s41598-018-34233-w. 30367154 10.1038/s41598-018-34233-w PMC6203800

[16] Ministério da Saúde. Brasil. Secretaria de Ciência, Tecnologia, Inovação e Insumos Estratégicos em Saúde. Relação Nacional de Medicamentos Essenciais Rename 2022. Available on: http://bvsms.saude.gov.br/bvs/publicacoes/relacao_nacional_medicamentos_2022.pdf.

[17] Ministério da Saúde. Brasil. Agência Nacional de Saúde Suplementar. Tabnet – Informações em saúde complementar: taxa de cobertura de planos de saúde. Available on: http://www.ans.gov.br/anstabnet/cgi-bin/dh?dados/ tabnet_tx.def.

[18] Ministério da Saúde. Brasil. Agência Nacional de Saúde Suplementar. Programas de Promoção da Saúde e Prevenção de Riscos e Doenças. Available on: https://www.ans.gov.br/index.php?option=com_promoprev&view=consulta

[19] Ministério da Saúde. Brasil. Agência Nacional de Saúde Suplementar. Tabnet – Informações em saúde complementar: Beneficiários por Operadora. Available on: https://www.ans.gov.br/anstabnet/cgi-bin/dh?dados/tabnet_cc.def7.

[20] Inker LA, Eneanya ND, Coresh J, Tighiouart H, Wang D, Sang Y, et al. Chronic Kidney Disease Epidemiology Collaboration. New Creatinine- and cystatin C-Based equations to Estimate GFR without Race. N Engl J Med. 2021;385(19):1737–49. 10.1056/NEJMoa2102953. 34554658 10.1056/NEJMoa2102953 PMC8822996

[21] Kidney Disease: Improving Global Outcomes (KDIGO) CKD Work Group. KDIGO clinical practice guideline for the evaluation and management of chronic kidney disease. Kidney Int Suppl. 2013;3:1-150.global outcomes 2012 clinical practice guideline. Ann Intern Med. 2013; 158(11):825 – 30. 10.7326/0003-4819-158-11-201306040-00007. 10.7326/0003-4819-158-11-201306040-00007 23732715

[22] de Sousa LCM, Silva NR, Azeredo CM, Rinaldi AEM, da Silva LS. Health-related patterns and chronic kidney disease in the Brazilian population: National Health Survey, 2019. Front Public Health. 2023;11:1090196. 10.3389/fpubh.2023.1090196. 37089474 10.3389/fpubh.2023.1090196 PMC10117670

[23] Samaan F, Damiani BB, Kirsztajn GM, Sesso R. A cross-sectional study on the prevalence and risk stratification of chronic kidney disease in Cardiological patients in São Paulo. Brazil Diagnostics (Basel). 2023;13(6):1146. 10.3390/diagnostics13061146. 36980454 10.3390/diagnostics13061146 PMC10047703

[24] Mazhar F, Sjölander A, Fu EL, Ärnlöv J, Levey AS, Coresh J, Carrero JJ. Estimating the prevalence of chronic kidney disease while accounting for nonrandom testing with inverse probability weighting. Kidney Int. 2023;103(2):416–20. 10.1016/j.kint.2022.10.027. 36462535 10.1016/j.kint.2022.10.027

[25] Feng T, Xu Y, Zheng J, Wang X, Li Y, Wang Y, Zhu B, Zhao L, Zhao H, Yu J. Prevalence of and risk factors for chronic kidney disease in ten metropolitan areas of China: a cross-sectional study using three kidney damage markers. Ren Fail. 2023;45(1):2170243. 10.1080/0886022X.2023.2170243. 36721891 10.1080/0886022X.2023.2170243 PMC9897755

[26] Navise NH, Mokwatsi GG, Gafane-Matemane LF, Fabian J, Lammertyn L. Kidney dysfunction: prevalence and associated risk factors in a community-based study from the North West Province of South Africa. BMC Nephrol. 2023;24(1):23. 10.1186/s12882-023-03068-7. 36717778 10.1186/s12882-023-03068-7 PMC9887915

[27] Guedes M, Rosa BB, Rocha PT, Teixeira CM, Pecoits-Filho R. Braz J Nephrol (J Bras Nefrol). 2022;44(3 Suppl 1):90. Limitações nas práticas de triagem e estratificação de risco da doença renal crônica no brasil: uma análise de um banco de dados laboratorial nacional.

[28] Gbaguidi GN, Houehanou CY, Amidou SA, Vigan J, Houinato DS, Lacroix P. Prevalence of abnormal kidney function in a rural population of Benin and associated risk factors. BMC Nephrol. 2021;22(1):116. 10.1186/s12882-021-02316-y. 33789608 10.1186/s12882-021-02316-y PMC8011182

[29] Vestergaard SV, Christiansen CF, Thomsen RW, Birn H, Heide-Jørgensen U. Identification of patients with CKD in Medical databases: a comparison of different algorithms. Clin J Am Soc Nephrol. 2021;16(4):543–51. 10.2215/CJN.15691021. 33707181 10.2215/CJN.15691021 PMC8092062

[30] Takeuchi M, Shinkawa K, Yanagita M, Kawakami K. Prevalence, recognition and management of chronic kidney disease in Japan: population-based estimate using a healthcare database with routine health checkup data. Clin Kidney J. 2021;14(10):2197–202. 10.1093/ckj/sfab016. 34676073 10.1093/ckj/sfab016 PMC8528067

[31] Jonsson AJ, Lund SH, Eriksen BO, Palsson R, Indridason OS. The prevalence of chronic kidney disease in Iceland according to KDIGO criteria and age-adapted estimated glomerular filtration rate thresholds. Kidney Int. 2020;98(5):1286–95. 10.1016/j.kint.2020.06.017. 32622831 10.1016/j.kint.2020.06.017

[32] Iwagami M, Tomlinson LA, Mansfield KE, Casula A, Caskey FJ, Aitken G, Fraser SDS, Roderick PJ, Nitsch D. Validity of estimated prevalence of decreased kidney function and renal replacement therapy from primary care electronic health records compared with national survey and registry data in the United Kingdom. Nephrol Dial Transplant. 2017; 1:32(suppl_2):ii142-ii150. 10.1093/ndt/gfw318. 10.1093/ndt/gfw318 PMC5410977 28201668

[33] Piccolli AP, Nascimento MM, Riella MC. Prevalence of chronic kidney disease in a population in southern Brazil (Pro-renal Study). Braz J Nephrol. 2017;39(4):384–90. 10.5935/0101-2800.20170070 29319764

[34] Benghanem Gharbi M, Elseviers M, Zamd M, Belghiti Alaoui A, Benahadi N, Trabelssi el H, Bayahia R, Ramdani B, De Broe ME. Chronic kidney disease, hypertension, diabetes, and obesity in the adult population of Morocco: how to avoid over- and under-diagnosis of CKD. Kidney Int. 2016;89(6):1363–71. 10.1016/j.kint.2016.02.019. 27165829 10.1016/j.kint.2016.02.019

[35] Ene-Iordache B, Perico N, Bikbov B, Carminati S, Remuzzi A, Perna A, Islam N, Bravo RF, Aleckovic-Halilovic M, Zou H, Zhang L, Gouda Z, Tchokhonelidze I, Abraham G, Mahdavi-Mazdeh M, Gallieni M, Codreanu I, Togtokh A, Sharma SK, Koirala P, Uprety S, Ulasi I, Remuzzi G. Chronic kidney disease and cardiovascular risk in six regions of the world (ISN-KDDC): a cross-sectional study. Lancet Glob Health. 2016;4(5):e307–19. 10.1016/S2214-109X(16)00071-1. 27102194 10.1016/S2214-109X(16)00071-1

[36] Galbraith LE, Ronksley PE, Barnieh LJ, Kappel J, Manns BJ, Samuel SM, Jun M, Weaver R, Valk N, Hemmelgarn BR. The see kidney disease targeted Screening Program for CKD. Clin J Am Soc Nephrol. 2016;11(6):964–72. 10.2215/CJN.11961115. 27197905 10.2215/CJN.11961115 PMC4891759

[37] Ji E, Kim YS. Prevalence of chronic kidney disease defined by using CKD-EPI equation and albumin-to-creatinine ratio in the Korean adult population. Korean J Intern Med. 2016;31(6):1120–30. 10.3904/kjim.2015.193. 27017386 10.3904/kjim.2015.193 PMC5094925

[38] Barreto SM, Ladeira RM, Duncan BB, Schmidt MI, Lopes AA, Benseñor IM, Chor D, Griep RH, Vidigal PG, Ribeiro AL, Lotufo PA, Mill JG. Chronic kidney disease among adult participants of the ELSA-Brasil cohort: association with race and socioeconomic position. J Epidemiol Community Health. 2016;70(4):380–9. 10.1136/jech-2015-205834. 26511886 10.1136/jech-2015-205834

[39] Pereira ERS, Pereira A, de Andrade C, de Naghettini GB, Pinto AV, Batista FKMS. Prevalência De doença renal crônica em adultos atendidos na Estratégia De Saúde Da Família. Braz J Nephrol. 2016;38(1):22–30. 10.5935/0101-2800.20160005. 10.5935/0101-2800.20160005

[40] Schaefer JCF, Pereira MS, Jesus CR, Schuelter-Trevisol F, Trevisol DJ. Estimativa Da função renal na população de 18 a 59 anos da cidade de Tubarão-SC: um estudo de base populacional. Braz J Nephrol. 2015;37(2):185–91.

[41] de Moura L, Andrade SSC, de Malta A, Pereira DC, Passos CA. Prevalência De autorrelato de diagnóstico médico de doença renal crônica no Brasil: Pesquisa Nacional De Saúde, 2013. Rev bras Epidemiol. 2015;18:181–91. 10.1590/1980-5497201500060016. 27008613 10.1590/1980-5497201500060016

[42] Dutra MC, Uliano EJM, Machado DFG, de Martins P, Schuelter-Trevisol T, Trevisol F. Avaliação Da função renal em idosos: um estudo de base populacional. Braz J Nephrol. 2014;36(3):297–303. 10.5935/0101-2800.20140043. 10.5935/0101-2800.20140043

[43] Castro AF, Coresh J. CKD surveillance using laboratory data from the population-based National Health and Nutrition Examination Survey (NHANES). Am J Kidney Dis. 2009;53(3 Suppl 3):S46–55. 10.1053/j.ajkd.2008.07.054. 19231761 10.1053/j.ajkd.2008.07.054 PMC2677815

[44] Bastos RMR, Bastos MG, Ribeiro LC, Bastos RV, Teixeira MTB. Prevalência Da doença renal crônica nos estágios 3, 4 e 5 em adultos. Revista Da Associação Médica Brasileira. 2009;55(1):40–4. 10.1590/S0104-42302009000100013. 19360276 10.1590/S0104-42302009000100013

[45] Fu EL, Coresh J, Grams ME, Clase CM, Elinder CG, Paik J, Ramspek CL, Inker LA, Levey AS, Dekker FW, Carrero JJ. Removing race from the CKD-EPI equation and its impact on prognosis in a predominantly white European population. Nephrol Dial Transpl. 2023;38(1):119–28. 10.1093/ndt/gfac197. 10.1093/ndt/gfac197 PMC9869854 35689668

[46] Delgado C, Baweja M, Burrows NR, Crews DC, Eneanya ND, Gadegbeku CA, Inker LA, Mendu ML, Miller WG, Moxey-Mims MM, Roberts GV, St Peter WL, Warfield C, Powe NR. Reassessing the inclusion of race in diagnosing kidney diseases: an Interim Report from the NKF-ASN Task Force. Am J Kidney Dis. 2021;78(1):103–15. 10.1053/j.ajkd.2021.03.008. 33845065 10.1053/j.ajkd.2021.03.008 PMC8238889

[47] Luyckx VA, Brenner BM. Low birth weight, nephron number, and kidney disease. Kidney Int Suppl. 2005;97S68–77. 10.1111/j.1523-1755.2005.09712.x. 10.1111/j.1523-1755.2005.09712.x 16014104

[48] Carrero JJ. Gender differences in chronic kidney disease: underpinnings and therapeutic implications. Kidney Blood Press Res. 2010;33(5):383–92. 10.1159/000320389. 20948227 10.1159/000320389

[49] Pereira M, Lunet N, Azevedo A, Barros H. Differences in prevalence, awareness, treatment and control of hypertension between developing and developed countries. J Hypertens. 2009;27(5):963–75. 10.1097/hjh.0b013e3283282f65. 19402221 10.1097/hjh.0b013e3283282f65

[50] Brasil. Ministério da Saúde. Secretaria de Vigilância em Saúde e Ambiente. Departamento de Análise Epidemiológica e Vigilância de Doenças Não Transmissíveis. Vigitel Brasil 2023: vigilância de fatores de risco e proteção para doenças crônicas por inquérito telefônico: estimativas sobre frequência e distribuição sociodemográfica de fatores de risco e proteção para doenças crônicas nas capitais dos 26 estados brasileiros e no Distrito Federal em 2023. Available on: http://bvsms.saude.gov.br/bvs/publicacoes/vigitel_brasil_2023.pdf.

