# Correction: Laboratory-based surveillance of chronic kidney disease in people with private health coverage in Brazil

**DOI:** 10.1186/s12882-024-03626-7

**Published:** 2024-07-19

**Authors:** Farid Samaan, Rubens Carvalho Silveira, Amilton Mouro, Gianna Mastroianni Kirsztajn, Ricardo Sesso

**Affiliations:** 1 Department of High Complexity Patients, Hapvida NotreDame Intermédica, Rua Hipódromo, 987 - Mooca, São Paulo, SP 03164-140 Brazil; 2 Secretaria de Estado da Saúde de São Paulo, São Paulo, SP Brazil; 3 Instituto Dante Pazzanese de Cardiologia, São Paulo, SP Brazil; 4 Faculdade de Saúde Pública, Universidade de São Paulo, São Paulo, SP Brazil; 5 Disciplina de Nefrologia, Universidade Federal de São Paulo, São Paulo, SP Brazil

**Correction: BMC Nephrology 25, 162 (2024)**

**https://doi.org/10.1186/s12882-024-03597-9**

Following publication of the original article [1], the authors identified some errors in Table 5. Commas were replaced with periods and values in the last line were corrected. Also, a few typos were corrected and the footnotes to figures 3 and 4, which were mistakenly inserted into the main text, were moved to the correct place.

The original article has been corrected.
