# Supplementary material for: Laboratory-based surveillance of chronic kidney disease in people with private health coverage in Brazil
Source: BMC Nephrol. 2024 May 10;25:162. doi: 10.1186/s12882-024-03597-9 (PMC11088147; doi:10.1186/s12882-024-03597-9)
Supplement: Supplementary file 1 — Supplementary Material 1 [file 12882_2024_3597_MOESM1_ESM.docx]

**Laboratory-based surveillance of chronic kidney disease in people with private health coverage in Brazil**

**Additional file 1**

Appendix Table 6S. Studies that evaluated the prevalence of chronic kidney disease.

| Author, year | Location | Population | No. | Age, years | Female, % | DM, % | Type of study | Evaluated exams | Definition of CKD | Prevalence of CKD, % | Gender with the highest prevalence | Other related factors |
| --- | --- | --- | --- | --- | --- | --- | --- | --- | --- | --- | --- | --- |
| de Souza^1^, 2023 | Brazil | PNS-2019 | 90,946 | 45 | 53 | 8 | Telephone survey | - | Self-reported diagnosis | 1.5% | No difference | Age, lower education level, Caucasian ethnicity, HT, DM, DLP, CVD, sedentary lifestyle |
| Samaan^2^, 2023 | Brazil | Adults at a hospital specializing in cardiology | 36,651 | 72 | 49 | - | Laboratory database | Single measurement of SCr | GFR <60 ml/min/1.73 m² (CKD-EPI 2009)^24^ | 30.9% | Female | Age |
| Mazhar^3^, 2023 | Sweden | Adult users of the public health system | 423,103 | 46 | 52 | 22 | Laboratory database | Single measurement of SCr and ACR | GFR <60 ml/min/1.73 m² (CKD-EPI 2009)^24^ or ACR >30 mg/g | 10.6% | Male | Age, HT, DM, CVD |
| Feng^4^, 2023 | China | Adults from 10 megacities, users of private insurance | 38,093 | 52 | 45 | 8 | Screening program | Single examination of SCr, ACR and urinary tract ultrasound | GFR <60 ml/min/1.73 m² (CKD-EPI 2009)^24^ or ACR >30 mg/g or alterations on ultrasound | 10.1% | Female | Age, HT, DM, hypertriglyceridemia, obesity, hyperuricemia |
| Navise^5^, 2023 | South Africa | PURE-study | 1,999 | 48 | 63 | 3 | Screening program | Single measurement of SCr and ACR | GFR <60 ml/min/1.73 m² (CKD-EPI 2009)^24^ or ACR >30 mg/g | 16.7% | Male | Age, dyslipidaemia, obesity, reactive protein-C |
| Guedes^6^, 2022 | Brazil | Adult users of supplementary health care | 4,523,332 | 48 | 60 | - | Laboratory database | Single measurement of SCr and ACR | GFR <60 ml/min/1.73 m² (CKD-EPI 2009)^24^ or ACR >30 mg/g | 10.8% | - | - |
| Gbaguidi^7^, 2021 | Benin | TAHES-study | 1,360 | 39 | 64 | 6 | Screening program | Single measurement of SCr | GFR <60 ml/min/1.73 m² (MDRD)^25^ | 16.1% | Female | Age, HT, DM, obesity |
| Vestergaard^8^, 2021 | Denmark | Adult users of the public health system | 84,688 | 74 | 53 | 10 | Laboratory database | Two SCr measurements with an interval of ≥90 days | GFR <60 ml/min/1.73 m² (CKD-EPI 2009)^24^ | 5.6% | - | - |
| Vestergaard^8^, 2021 | Denmark | Adult users of the public health system | 100,957 | 73 | 51 | 12 | Laboratory database | Two SCr measurements and two ACR measurements with an interval ≥90 days | Duas GFR <60 ml/min/1.73 m² (CKD-EPI 2009)^24^ or duas ACR >30 mg/g | 5.9% | - | - |
| Vestergaard^8^, 2021 | Denmark | Adult users of the public health system | 27,947 | 72 | 42 | 17 | Electronic medical records of patients | - | Record of diagnosis in medical records | 0.8% | - | - |
| Vestergaard^8^, 2021 | Denmark | Adult users of the public health system | 103,435 | 71 | 51 | 8 | Laboratory database | Single measurement of SCr | GFR <60 ml/min/1.73 m² (CKD-EPI 2009)^24^ | 8.3% | - | - |
| Takeushi^9^, 2021 | Japan | Active workers and their families with private health insurance | 761,565 | 56 | 31 | 10 | Laboratory database | Single measurement of SCr | GFR <60 ml/min/1.73 m² (specific equation for the Japanese population) ^26^ | 7.2% | - | - |
| Jonsson^10^, 2020 | Iceland | Adult users of the public health system | 218,437 | 63 | 53 | 13 | Laboratory database | Two SCr measurements and two ACR measurements with an interval ≥90 days | Duas GFR <60 ml/min/1.73 m² (CKD-EPI 2009)^24^ or duas ACR >30 mg/g | 5.9% | Female | - |
| Iwagami^11^, 2017 | United Kingdom | Adult users of the public health system | 2,761,755 | 53 | 51 | - | Laboratory database | Single measurement of SCr | GFR <60 ml/min/1.73 m² (CKD-EPI 2009)^24^ | 6.9% | Female | Age |
| Picolli^12^, 2017 | Brazil | Adult employees of an energy company, Paraná | 5,216 | 45 | 64 | 7 | Screening program | Single measurement of SCr and proteinuria (reagent strip) | GFR <60 ml/min/1.73 m² (MDRD) ^25^ or proteins equal to or above 1+ | 11.5% | Female | Age, HT, DM, obesity |
| Gharbi^13^, 2016 | Morocco | MAREMAR-study (2 cities) | 10,524 | 48 | 51 | 13 | Population study | Two SCr measurements and two ACR measurements with an interval ≥90 days | Two GFRs <60 ml/min/1.73 m² (CKD-EPI 2009^24^ or duas ACR >30 mg/g | 5.1% | No difference | Age, HT, DM, obesity |
| Ene-Iordaque^14^, 2016 | 12 countries, 6 regions of the world | Adult users of the public health system | 75,058 | 45 | 38 | 71 | Screening program | Single measurement of SCr and proteinuria (reagent strip or ACR) | GFR <60 ml/min/1.73 m² (CKD-EPI 2009)^24^ or proteins ≥ 1+ or ACR >30 mg/g | 14.3% | Female | HT, DM, DLP, obesity |
| Galbraith^15^, 2016 | Canada | Adults with risk factors for CKD | 5,144 | 59 | 65 | 23 | Screening program | Single measurement of SCr | GFR <60 ml/min/1.73 m² (CKD-EPI 2009)^24^ | 18.8% | Female | HT, DM, obesity |
| Ji1^16^, 2016 | South Korea | KNHANES 2010-2012 | 10,636 | 46 | 55 | 9 | Population study | Single measurement of SCr and ACR | GFR <60 ml/min/1.73 m² (CKD-EPI 2009)^24^ or ACR >30 mg/g | 7.9% | Female | Age, HT, DM, DLP, obesity |
| Barreto^17^, 2016 | Brazil | ELSA-Brazil (6 capitals) | 14,636 | 52 | 54 | 20 | Screening program | Single measurement of SCr and proteinuria (reagent strip or ACR) | GFR <60 ml/min/1.73 m² (CKD-EPI 2009)^24^ or ACR >30 mg/g | 8.9% | No difference | Age, lower education level, black, mixed-race and indigenous ethnicities, HT, DM, DLP, CVD, obesity, smoking |
| Pereira^18^, 2015 | Brazil | Adult users of the public health system, Goiás | 511 | 45 | 68 | 12 | Screening program | Single measurement of SCr and proteinuria (reagent strip or ACR) | GFR <60 ml/min/1.73 m² (Cockroft-Gault)^27^ or ACR >30 mg/g | 32.5% | Male | Age, DM, alcohol consumption |
| Shaefer^19^, 2015 | Brazil | Users of the public health system, aged between 18-59 years, Tubarão, SC | 371 | 40 | 64 | 6 | Population study | Single measurement of SCr and ACR | GFR <60 ml/min/1.73 m² (MDRD)^25^ or ACR >30 mg/g | 1.4% | No difference | Obesity |
| de Moura^20^, 2015 | Brazil | PNS-2013 | 60,202 | - | - | - | Telephone survey | - | Self-reported diagnosis | 1.4% | No difference | Age, lower education level, South and Midwest regions |
| Dutra^21^, 2014 | Brazil | Users of the public health system, aged 60 years or older, residents of Tubarão, SC | 822 | 69 | 62 | 24 | Population study | Single measurement of SCr | GFR <60 ml/min/1.73 m² (CKD-EPI 2009)^24^ | 13.6% | No difference | Age, lower education level, South and Midwest regions of the city |
| Castro^22^, 2009 | United States | NHANES 1999-2004 | 13,233 | 46 | 47 | 7 | Population study | Single measurement of SCr and ACR | GFR <60 ml/min/1.73 m² (MDRD)^25^ or ACR >30 mg/g | 13.1% | Female | Age, white ethnicity, HT, DM |
| Castro^22^, 2009 | United States | NHANES 1988-1994 | 15,488 | 45 | 52 | 5 | Population study | Single measurement of SCr | GFR <60 ml/min/1.73 m² (MDRD)^25^ or ACR >30 mg/g | 10.0% | Female | Age, white ethnicity, HT, DM |
| Bastos^23^, 2009 | Brazil | Adult users of private health care in Juiz de Fora, MG | 24,248 | 48 | 60 | - | Laboratory database | Single measurement of SCr and ACR | GFR <60 ml/min/1.73 m² (MDRD)^25^ | 12.4% | Female | Age |

DM, diabetes mellitus. CKD, chronic kidney disease. HT, hypertension. DM, diabetes mellitus. DLP, dyslipidemia. CVD, cardiovascular disease. SCr, serum creatinine. GFR, estimated glomerular filtration rate. CKD-EPI, Chronic Kidney Disease Epidemiology Collaboration. ACR, albumin/creatinine ratio in an isolated urine sample. MDRD, Modification of Diet in Renal Disease. PURE-study, Prospective Urban Rural Epidemiology. TAHES, Tanve Health Study. PNS, Plano Nacional de Saúde. KNHANES, Korea National Health and Nutrition Examination Survey. ELSA-Brasil, Estudo Longitudinal de Saúde do Adulto. MAREMAR, Maladies rénales chroniques au Maroc. NHANES, National Health and Nutrition Examination Survey.

**References**

1. de Sousa LCM, Silva NR, Azeredo CM, Rinaldi AEM, da Silva LS. Health-related patterns and chronic kidney disease in the Brazilian population: National Health Survey, 2019. Front Public Health. 2023; 11:1090196. doi: 10.3389/fpubh.2023.1090196
2. Samaan F, Damiani BB, Kirsztajn GM, Sesso R. A Cross-Sectional Study on the Prevalence and Risk Stratification of Chronic Kidney Disease in Cardiological Patients in São Paulo, Brazil. Diagnostics (Basel). 2023 Mar 16;13(6):1146. doi: 10.3390/diagnostics13061146
3. Mazhar F, Sjölander A, Fu EL, Ärnlöv J, Levey AS, Coresh J, Carrero JJ. Estimating the prevalence of chronic kidney disease while accounting for nonrandom testing with inverse probability weighting. Kidney Int. 2023 Feb;103(2):416-420. doi: 10.1016/j.kint.2022.10.027
4. Feng T, Xu Y, Zheng J, Wang X, Li Y, Wang Y, Zhu B, Zhao L, Zhao H, Yu J. Prevalence of and risk factors for chronic kidney disease in ten metropolitan areas of China: a cross-sectional study using three kidney damage markers. Ren Fail. 2023; 45(1):2170243. doi: 10.1080/0886022X.2023.2170243
5. Navise NH, Mokwatsi GG, Gafane-Matemane LF, Fabian J, Lammertyn L. Kidney dysfunction: prevalence and associated risk factors in a community-based study from the North West Province of South Africa. BMC Nephrol. 2023; 24(1):23. doi: 10.1186/s12882-023-03068-7
6. Guedes M, Rosa BB, Rocha PT, Teixeira CM, Pecoits-Filho R. Limitações nas práticas de triagem e estratificação de risco da doença renal crônica no brasil: uma análise de um banco de dados laboratorial nacional. Braz. J. Nephrol. (J. Bras. Nefrol.) 2022;44(3 Suppl 1):90
7. Gbaguidi GN, Houehanou CY, Amidou SA, Vigan J, Houinato DS, Lacroix P. Prevalence of abnormal kidney function in a rural population of Benin and associated risk factors. BMC Nephrol. 2021; 22(1):116. doi: 10.1186/s12882-021-02316-y
8. Vestergaard SV, Christiansen CF, Thomsen RW, Birn H, Heide-Jørgensen U. Identification of Patients with CKD in Medical Databases: A Comparison of Different Algorithms. Clin J Am Soc Nephrol. 2021; 16(4):543-551. doi: 10.2215/CJN.15691021
9. Takeuchi M, Shinkawa K, Yanagita M, Kawakami K. Prevalence, recognition and management of chronic kidney disease in Japan: population-based estimate using a healthcare database with routine health checkup data. Clin Kidney J. 2021; 14(10):2197-2202. doi: 10.1093/ckj/sfab016
10. Jonsson AJ, Lund SH, Eriksen BO, Palsson R, Indridason OS. The prevalence of chronic kidney disease in Iceland according to KDIGO criteria and age-adapted estimated glomerular filtration rate thresholds. Kidney Int. 2020; 98(5):1286-1295. doi: 10.1016/j.kint.2020.06.017
11. Iwagami M, Tomlinson LA, Mansfield KE, Casula A, Caskey FJ, Aitken G, Fraser SDS, Roderick PJ, Nitsch D. Validity of estimated prevalence of decreased kidney function and renal replacement therapy from primary care electronic health records compared with national survey and registry data in the United Kingdom. Nephrol Dial Transplant. 2017; 1:32(suppl_2):ii142-ii150. doi: 10.1093/ndt/gfw318
12. Piccolli AP, Nascimento MM, Riella MC. Prevalence of chronic kidney disease in a population in southern Brazil (Pro-Renal Study). Braz. J. Nephrol. 2017;39(4):384-90.
13. Benghanem Gharbi M, Elseviers M, Zamd M, Belghiti Alaoui A, Benahadi N, Trabelssi el H, Bayahia R, Ramdani B, De Broe ME. Chronic kidney disease, hypertension, diabetes, and obesity in the adult population of Morocco: how to avoid "over"- and "under"-diagnosis of CKD. Kidney Int. 2016; 89(6):1363-71. doi: 10.1016/j.kint.2016.02.019
14. Ene-Iordache B, Perico N, Bikbov B, Carminati S, Remuzzi A, Perna A, Islam N, Bravo RF, Aleckovic-Halilovic M, Zou H, Zhang L, Gouda Z, Tchokhonelidze I, Abraham G, Mahdavi-Mazdeh M, Gallieni M, Codreanu I, Togtokh A, Sharma SK, Koirala P, Uprety S, Ulasi I, Remuzzi G. Chronic kidney disease and cardiovascular risk in six regions of the world (ISN-KDDC): a cross-sectional study. Lancet Glob Health. 2016; 4(5):e307-19. doi: 10.1016/S2214-109X(16)00071-1
15. Galbraith LE, Ronksley PE, Barnieh LJ, Kappel J, Manns BJ, Samuel SM, Jun M, Weaver R, Valk N, Hemmelgarn BR. The See Kidney Disease Targeted Screening Program for CKD. Clin J Am Soc Nephrol. 2016; 11(6):964-972. doi: 10.2215/CJN.11961115
16. Ji E, Kim YS. Prevalence of chronic kidney disease defined by using CKD-EPI equation and albumin-to-creatinine ratio in the Korean adult population. Korean J Intern Med. 2016; 31(6):1120-1130. doi: 10.3904/kjim.2015.193
17. Barreto SM, Ladeira RM, Duncan BB, Schmidt MI, Lopes AA, Benseñor IM, Chor D, Griep RH, Vidigal PG, Ribeiro AL, Lotufo PA, Mill JG. Chronic kidney disease among adult participants of the ELSA-Brasil cohort: association with race and socioeconomic position. J Epidemiol Community Health. 2016; 70(4):380-9. doi: 10.1136/jech-2015-205834
18. Pereira ERS, Pereira A de C, Andrade GB de, Naghettini AV, Pinto FKMS, Batista SR, et al.Prevalência de doença renal crônica em adultos atendidos na Estratégia de Saúde da Família. Braz J Nephrol. 2016; 38(1):22–30. doi: 10.5935/0101-2800.20160005
19. Schaefer JCF, Pereira MS, Jesus CR, Schuelter-Trevisol F, Trevisol DJ. Estimativa da função renal na população de 18 a 59 anos da cidade de Tubarão-SC: Um estudo de base populacional. Braz. J. Nephrol. 2015; 37(2):185-91.
20. Moura L de, Andrade SSC de A, Malta DC, Pereira CA, Passos JEF. Prevalência de autorrelato de diagnóstico médico de doença renal crônica no Brasil: Pesquisa Nacional de Saúde, 2013. Rev bras epidemiol. 2015; 18:181–91. doi: 10.1590/1980-5497201500060016
21. Dutra MC, Uliano EJM, Machado DFG de P, Martins T, Schuelter-Trevisol F, Trevisol DJ. Avaliação da função renal em idosos: um estudo de base populacional. Braz J Nephrol. 2014; 36(3):297–303. doi: 10.5935/0101-2800.20140043
22. Castro AF, Coresh J. CKD surveillance using laboratory data from the population-based National Health and Nutrition Examination Survey (NHANES). Am J Kidney Dis. 2009; 53(3 Suppl 3):S46-55. doi: 10.1053/j.ajkd.2008.07.054
23. Bastos RMR, Bastos MG, Ribeiro LC, Bastos RV, Teixeira MTB. Prevalência da doença renal crônica nos estágios 3, 4 e 5 em adultos. Revista Da Associação Médica Brasileira, 2009; 55(1), 40–44. doi: 10.1590/S0104-42302009000100013
24. Levey AS, Stevens LA, Schmid CH, Zhang YL, Castro AF 3rd, Feldman HI, Kusek JW, Eggers P, Van Lente F, Greene T, Coresh J; CKD-EPI (Chronic Kidney Disease Epidemiology Collaboration). A new equation to estimate glomerular filtration rate. Ann Intern Med. 2009;150(9):604-12. doi: 10.7326/0003-4819-150-9-200905050-00006
25. Levey AS, Bosch JP, Lewis JB, Greene T, Rogers N, Roth D. A more accurate method to estimate glomerular filtration rate from serum creatinine: a new prediction equation. Modification of Diet in Renal Disease Study Group. Ann Intern Med. 1999;130(6):461-70. doi: 10.7326/0003-4819-130-6-199903160-00002
26. Horio M, Imai E, Yasuda Y, Watanabe T, Matsuo S. Modification of the CKD epidemiology collaboration (CKD-EPI) equation for Japanese: accuracy and use for population estimates. Am J Kidney Dis. 2010; 56(1):32-8. doi: 10.1053/j.ajkd.2010.02.344
27. Cockcroft DW, Gault MH. Prediction of creatinine clearance from serum creatinine. Nephron 1976; 16:31-41.
