# Supplementary material for: Laboratory-based surveillance of chronic kidney disease in people with private health coverage in Brazil
Source: BMC Nephrol. 2024 May 10;25:162. doi: 10.1186/s12882-024-03597-9 (PMC11088147; doi:10.1186/s12882-024-03597-9)
Supplement: Supplementary file 2 — Supplementary Material 2 [file 12882_2024_3597_MOESM2_ESM.docx]

**Laboratory-based surveillance of chronic kidney disease in people with private health coverage in Brazil**

**Additional file 2**

Appendix Table 7S. Distribution of study participants in the GFR ranges according to the 2009 and 2021 CKD-EPI equations.

| GFR  (ml/min/1.73 m²) | CKD-EPI equation | | | | |
| --- | --- | --- | --- | --- | --- |
|  | 2009 | |  | 2021 | |
|  | Number | Proportion (%) |  | Number | Proportion (%) |
| ≥90 | 905,915 | 60.04 |  | 1,018,029 | 67.47 |
| 60-89 | 526,450 | 34.89 |  | 433,523 | 28.73 |
| < 60 | 76,401 | 5.06 |  | 57,214 | 3.79 |
| 45-59 | 53,287 | 3.53 |  | 38,888 | 2.58 |
| 30-44 | 16,054 | 1.06 |  | 12,276 | 0.81 |
| 15-29 | 4,501 | 0.30 |  | 3,652 | 0.24 |
| < 15 | 2,559 | 0.17 |  | 2,398 | 0.16 |
| Total | 1,508,766 | 100.00 |  | 1,508,766 | 100.00 |

GFR, glomerular filtration rate. CKD-EPI, Chronic Kidney Disease Epidemiology Collaboration.
